# Wear simulation of UHMWPE against the different counterface roughness in reciprocating unidirectional sliding motion

**DOI:** 10.1038/s41598-024-66613-w

**Published:** 2024-07-09

**Authors:** Chen Ting, Ma Weiguo, Zhu Kaihui, Hu Zilong

**Affiliations:** 1 School of Mechanical Engineering, Yangtze University, Jingzhou, 434023 China; 2 SJS Petroleum Drilling & Production Equipment Co. LTD, Jingzhou, 434023 China

**Keywords:** UHMPE, Wear models, Finite element method, Counterface, Roughness, Engineering, Materials science

## Abstract

Wear simulations of UHMWPE can economically and conveniently predict the performance of wear resistant bushings used for sealing or other reciprocating unidirectional sliding motion. In this study, pin on plate tribological experiments and microscopic analysis was done to obtained the wear profiles, wear volume and wear mechanism of UHMWPE against the counterface with different surface roughness of which Ra range is 0.03 ~ 2 μm. Meanwhile, the 3D wear simulation model of the pin on plate tribological experiments was established to discuss the adaptability of the energy and Archard wear model by analyzing the difference of wear profiles and wear volume between the experiment and simulation. The results indicate that with an increase in the counterface roughness, the wear simulation of UHMWPE estimated by the energy model were more accurate in reciprocating unidirectional sliding motion.

## Introduction

Ultrahigh molecular weight polyethylene (UHMWPE) is a special polymer material with excellent physical and chemical properties, such as chemical inertness, self-lubrication, impact resistance, wear-resistance and abrasion resistance^1^, which has been the preferred material used as wear resistant for seals^2^, water lubricated bearing material bearing^3^, medical devices (artificial hips) ^1^ or others sliding elements^4^. However, wear is still an important factor that affects the life cycle of UHMWPE components^4^. Especially for the seals, wear of UHMWPE components may lead to safety, and the movement form is mostly reciprocating unidirectional sliding^2^. Therefore, the knowledge of the wear evolution law and the effective life of UHMWPE seal components in reciprocating unidirectional sliding motion have a positive impact on its design.

Research on the friction and wear behaviors of UHMWPE mainly focuses on by standard experimental methods, while it cannot reflect well on the evolution of wear and the effective life of UHMWPE components, and it may be necessary to perform full-scale experiments in the real working conditions^5^. Nevertheless, the full-scale experiments require significant manpower and material resources. With the rapid development of computer science, the wear law of the materials can be predicted by finite element method, which is convenient to investigate the wear performance and evolution during working processes^6^. To date, the user-defined subroutine UMESHMOTION in the commercial finite element package ABAQUS is a common tool to realize the wear simulation based on finite element method, which can obtain reasonable results for wear simulation as long as the wear model is accurate^5^.

Scholars have done some research on wear simulation of UHMWPE component. Bevill et al.^7^ established the wear simulation of hip replacement articulation (UHMWPE) to study the interaction of creep and wear, and the wear model is Archard model. S. O’Brien^8^ developed a computational model which is a combination of the wear simulation model based on Archard’s wear model and wear volume distribution formula to predict the tibial insert articular and backside surfaces (UHMWPE) wear in modular total knee replacements. Kang et al.^9^ established the tibial inserts of UHMWPE wear simulation model based on Archard model to predict its wear when different surface treatment methods (the wear factor and coefficients of friction are used to characterize surface treatment method), and the rationality of the simulation model is verified compared with the experimental results. Recent studies have shown that UHMWPE wear under multidirectional sliding motion is susceptible to cross-shear (CS), it's been proven that the Archard model loses fidelity in multidirectional sliding motion of the UHMWPE^10–12^. Kang et al.^13^ improved Archard model including the effects of cross shear and contact pressure, and carried out wear simulation of UHMWPE hip implants. Feng et al.^14^ build a new wear model of UHMWPE that by incorporating the energy dissipation and contact pressure in quantifying the CS effect and wear. In conclusion, researches on UHMWPE wear simulation in literature mainly focus on the influence of contact pressure, sliding mode (CS) and energy dissipation on wear simulation.

Furthermore, the counterface roughness has a great influence on tribology behaviors of UHMWPE, and some researches have indicated it by Tribological experiment. Lloyd et al.^15^ investigated the effect of counterface surface roughness (AISI 431) on the wear of UHMWPE with a reciprocating sliding wear rig, the surface roughness Ra range is 0.1–1.0 μm, and the results show that counterface surface roughness directly affects the wear mechanisms and wear factor of UHMWPE. M.E. et al.^16^ studied the effect of counterface roughness (CoCr) on the cross wear of UHMWPE through multi-directional wear testing, the surface roughness Ra value is 0.015 and 0.45 μm, and the results showed that the counterface roughness had a greater impact on the wear factor in the rectangular wear path with large length-to-diameter ratio. V Saikko et al.^17,18^ analyzed the effect of counterface roughness (CoCr) on the wear of conventional gamma-sterilized and electron-beam-crosslinked UHMWPE with a circularly translating pin-on-disk (POD) tester and the Noncyclic RandomPOD tester respectively, the surface roughness Ra range is 0.014 ~ 0.24 μm, and the results indicated that crosslinking could improve wear resistance and wear factor of UHMWPE is strongly correlated with Ra. However, so far, the research about discussing the adaptability of the wear models in finite element method to predict the wear of UHMWPE against the counterface with different surface roughness has not been reported. In the existing studies, the counterface roughness value is mainly discussed at 0.014 ~ 1 μm, but the research results may not be applicable in harsh working conditions, such as oil wellhead seals.

The energy model and Archard model are often used in wear simulations under various working conditions and motion forms^19–22^. This study intends to establish the 3D finite element model to simulate UHMWPE wear and to discuss the adaptability of the energy model and Archard model for predicting the wear of UHMWPE against the different counterface roughness in reciprocating unidirectional sliding motion. Besides, friction and wear behavior of UHMWPE against different countersurface roughness under dry condition were investigated by a pin-on-plate tribometer, and the study range of the counterface roughness increases to 0.03 ~ 2 μm in this paper. The corresponding wear coefficients of the energy model and Archard model were obtained by the experimental data, the corresponding finite element models for UHMWPE pin wear in a pin-on-plate tribometer were established using the user-defined subroutine, UMESHMOTION, in the commercial finite element package ABAQUS (V6.14, Dassault System, France). The adaptability of the two models was investigated by comparing the wear profiles and the wear volume predicted from finite element method with the experimental data, which will provide a theoretical basis for the selection of the wear simulation model of UHMWPE in reciprocating unidirectional sliding motion, especially for the wear prediction of UHMWPE seals in the reciprocating unidirectional sliding under harsh working conditions.

## Experimental data and wear model

### Tribological tests

#### Materials

Tribological tests of UHMWPE were conducted on a reciprocating pin-on-plate tribometer. The material and geometric parameters of pin and plate are shown in Table 1. According to the ASTM G99-04 specification, the pin is cylindrical with a 4-mm diameter and 20-mm length and the plates are rounded with a 25-mm diameter and 2-mm thickness, which were machined. Before tests, the plates were polished using sandpaper that contained 400, 150, and 60 mesh and then with diamond spray polishes, making its surface roughness Ra up to 0.03, 0.75 and 2.04 μm, respectively. In this paper, to obtain an accurate surface roughness, six different positions of the plate surface were measured with a JB-5C stylus profilometer, and each position was measured twice after the average value of surface roughness Ra reached 0.03, 0.75 and 2.04 μm. Finally, the pins and plates were rinsed with ethanol for 15 min, were blown dry, and then were placed in the dryer for use.

**Table 1:** The main composition of the carbon steel.

Test sample	Pin		Plate
Material	TECAFINEPE 10	Q235	
Diameter[mm]	4	25	
Pin length[mm]	20	_	
Plate thickness[mm]	_	2	
Ra surface roughness[μm]	_	0.03 0.75 2.04	

#### Wear test

**Figure 1:**
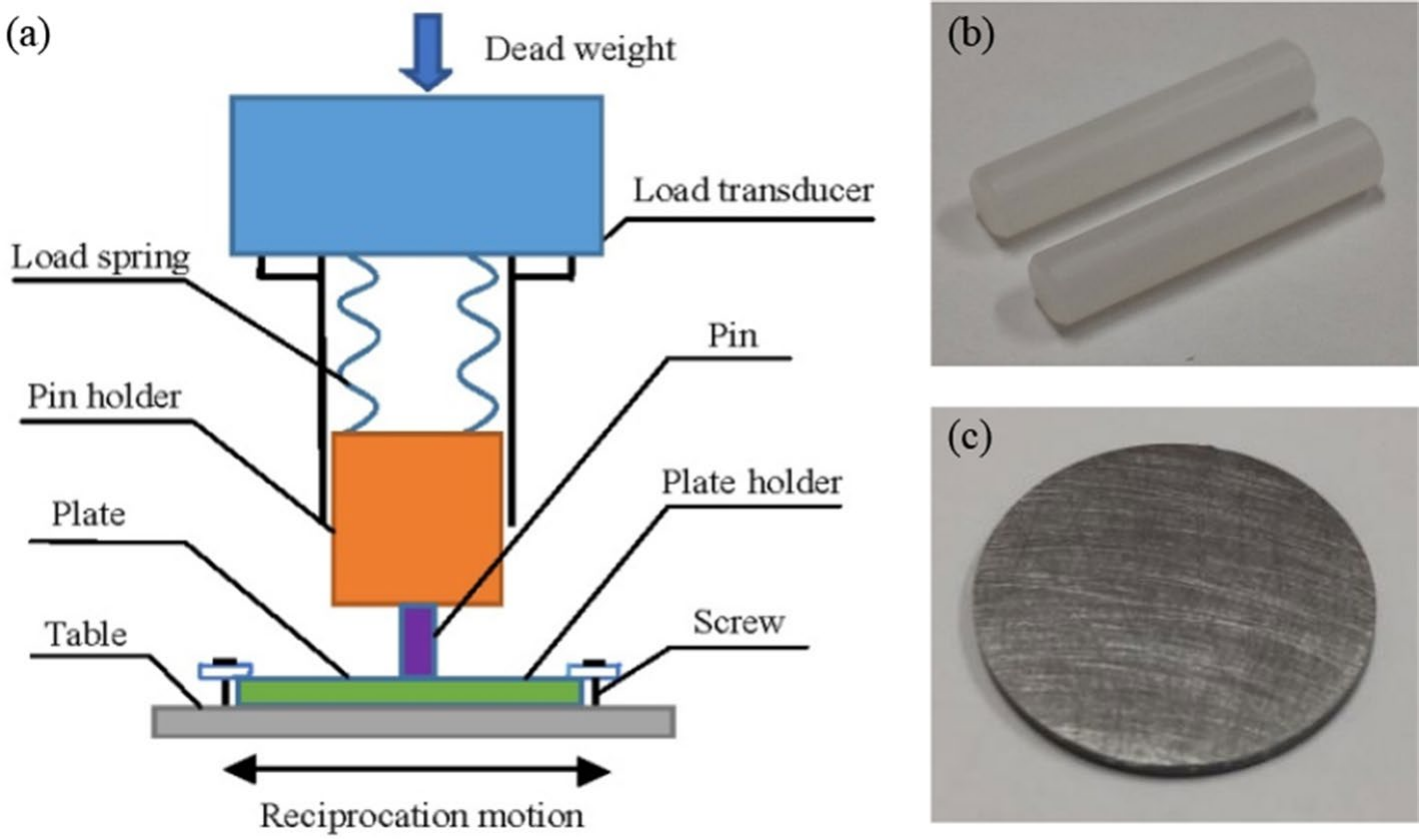
Scheme of the pin-on-plate tribometer (**a**) with the UHMWPE pin (**b**) and plate (**c**) images.

The tribological tests was carried out by the pin-on-plate tribometer under a laboratory condition of that temperature is 23℃ and the humidity is 40%. The schematic of the pin-on-plate tribometer is shown in Fig. 1. According to the ASTM G99-04 specification, UHMWPE pins and plates were attached to the sample holder and the plate continuously ran with reciprocating motion, the normal load was set to 60 N, the test speed was 100 mm/s, the stroke range was 5 mm, and reciprocating frequency is 10 Hz. The test time was 60 min, which corresponded to a total cumulative slip distance of 360,000 mm. Each set tests were repeated five times, and each test uses a new pin and plate sample. The mass of the sample was measured by an electronic balance. Scanning electron microscope (JSM-IT300, JEOL, Japan) and laser microscope (KEYENCE VK-X200k, Keyence Corporation, Japan) were used to characterize the microscopic surface morphology to analyze the wear mechanism of the UHMWPE. The results of wear test were shown in section "Experimental results and Finite element model".

#### Experimental results

**Figure 2:**
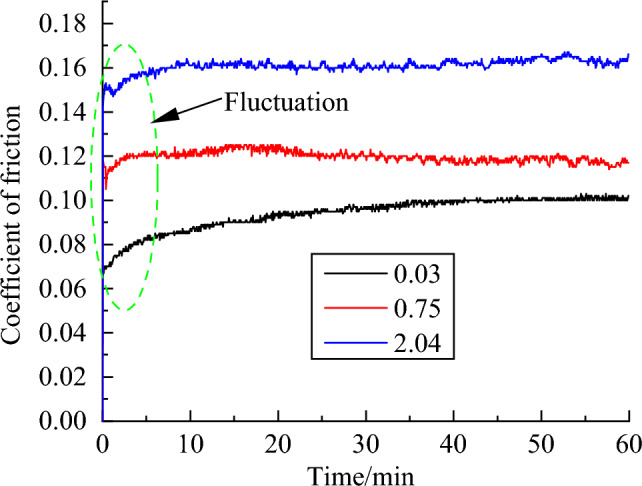
Coefficient of friction of UHMWPE against plates with different surface roughness.

**Figure 3:**
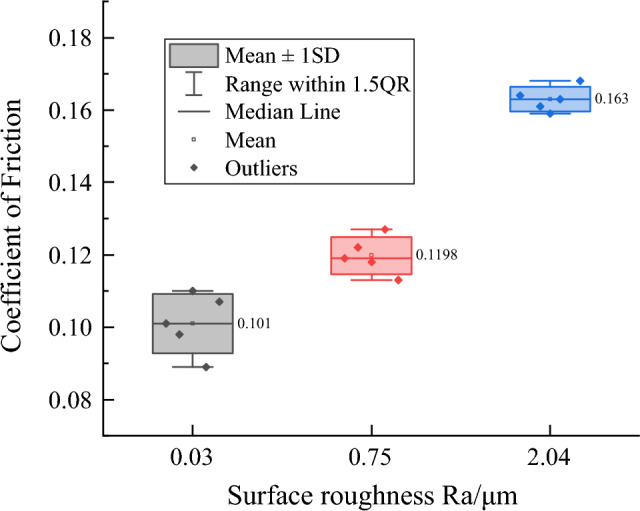
The coefficient of friction over the entire test period of 60 min.

Figure 2 depicts the variation of the coefficient of friction of a UHMWPE pin with a surface roughness of 0.03, 0.75 and 2.04 μm. As seen in Fig. 2, the coefficient of friction gradually increased along with test time increasing, and then the coefficient of friction remained stabilized. It is to be found that the fluctuations were significant within 10 min, which is in the running-in stage. This result could be explained by the material transfer being involved at the running-in stage, which induces to a greater wear rate at this stage, and this phenomenon indicates the presence of morphological changes in the contact between the mating materials^17,18,23^. After the entire test period of 60 min, the average coefficient of friction that the UHMWPE pin is against the plate with different surface roughness is shown in Fig. 3. It can be clearly observed that the coefficient of friction increased with surface roughness increasing in the plate.

Furthermore, the dissipation of energy that the work of friction force produced can cause the surface temperature of the UHMWPE pin and steel plate to rise, which is the main cause of geometric and chemical modifications under dry conditions^16^. Therefore, the rise of the coefficient of friction can be related to severe plastic deformation and/or adherence of polymer in the contact^23,24^. After the entire test period of 60 min, the average coefficient of friction that the UHMWPE pin is against the plate with different surface roughness is shown in Fig. 3. It can be clearly observed that the coefficient of friction increased with surface roughness increasing in the plate.

**Figure 4:**
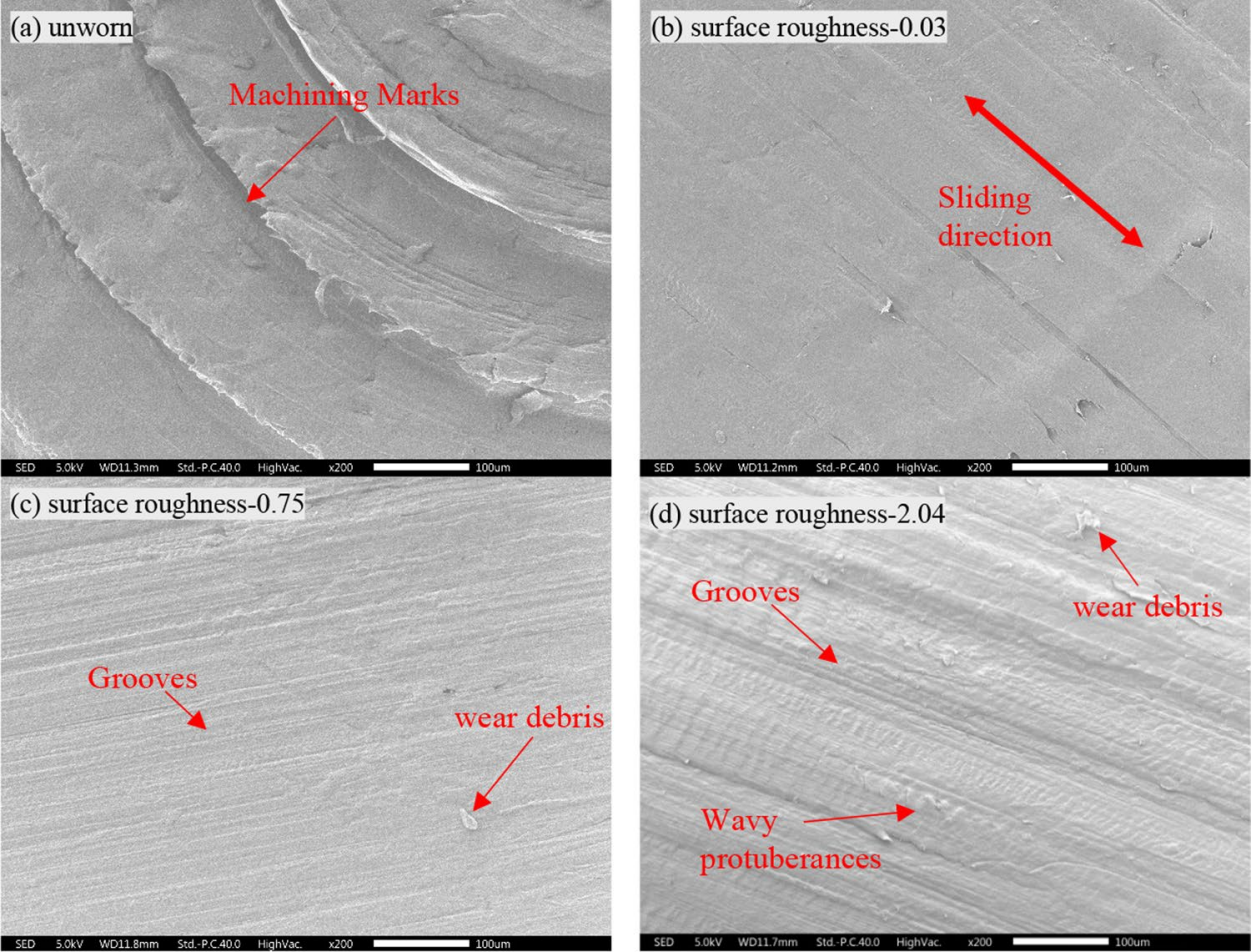
SEM images of an unworn (**a**) and worn UHMWPE pin against plate with Ra of 0.03 μm (**b**), 0.75 μm (**c**) and 2.04 μm (**d**) after 60 min wear test. The arrow represents the sliding direction.

To characterize the tribology of UHMWPE more comprehensively, the worn surface of a UHMWPE pin after a 60 min wear test was investigated using a scanning electron microscope, and the SEM images is Fig. 4 shows the influence of the counterface roughness on the worn surface of UHMWPE. As seen from Fig. 4b, when Ra is 0.03 μm, the worn surface of the UHMWPE pin suffered some damage: some grooves with different depths in parallel to the reciprocating sliding direction, which may be attributed to the abrasive wear of micro-cutting and micro-plowing^25^. In addition, with contrast to the SEM images of the unworn UHMWPE pin Fig. 4a, it can be found that some initial machining marks exist. It may be further speculated that the wear volume of the UHMWPE pin is minimal. When Ra is 0.75 μm (Fig. 4c), there were some grooves and a small number of wavy protuberances perpendicular to the sliding direction, and when Ra increases to 2.04 μm (Fig. 4d), a large number of wavy protuberances occurs with a clear appearance of delamination. It can be found that as the counterface roughness increases, the number of wavy protuberances increases. This finding is most likely observed because the surface asperities act on the UHMWPE pin surface layer to form a high-pressure stress zone with the Ra increasing, causing the surface material to have plastic deformation and then to form protuberances^23,24^. Moreover, repeated sliding contact friction led to the contact temperatures to elevate and form wavy protuberances, and this leads to a higher coefficient of friction observed in Fig. 3^16^. This also supposes the surface fatigue wear at the interface of the pin^26,27^. With the increase in the Ra, the wear mechanism of UHMWPE becomes more complex.

### Tensile test

**Figure 5:**
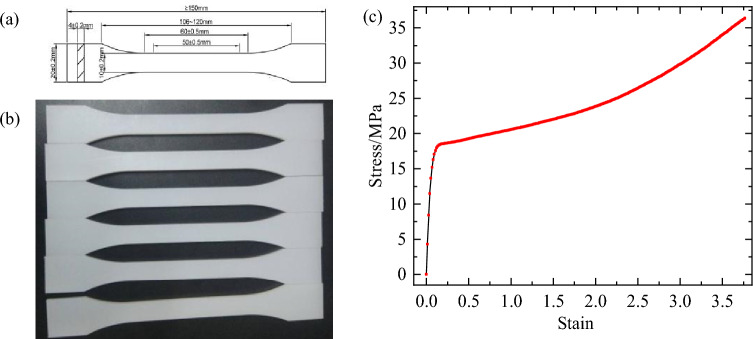
Specimen size (**a**), specimen (**b**) and the stress–strain curves (**c**).

For accurately characterizing the mechanical behavior of UHMWPE and obtaining the stress–strain curve, the tensile test was carried out in an ambient room environment. According to the ISO 527–1:1993 standard, the tensile test repeated five times was conducted at the speed of 50 mm/min; a П type specimen was selected, and its structural sizes are shown in Fig. 5a-b. During the tensile test, the force–displacement was continuously monitored until the specimen was torn, and it was valid only when the break location of the specimen was in the narrow part. Finally, the stress–strain curve was converted from the force–displacement curves obtained by averaging the test data, as shown in Fig. 5c.

### Wear model

#### Archard model

The Archard model is widely used for finite element model to simulate wear^28–30^, which is a simple phenomenological model that assumes a proportionality relationship between the wear volume, which is expressed as

1 $$ V = k\frac{FL}{H} $$

where *V* is the wear volume in mm^3^, *L* is the total accumulated slip distance in mm, *F* is the normal load applied on the disc surface in N, and $H$ is the hardness of the soft material in MPa, *k* is the dimensionless wear coefficients, which can be obtained dependent on the experimental results. Equation (1) can be divided by a contact area,

2 $$ \frac{dV}{{dLdA}} = k\frac{dF}{{HdA}} $$

$dF/dA$ where the local contact pressure, $dV/dA$ is the local wear depth, $dh$. Equation (2) can be re-written as Eq. (3) to implement in 3D FEA models.

3 $$ dh = k_{A} p(x)dL $$

where $p(x)$ is the local normal contact stress in computational domain computed by the contact algorithmic of the software of finite element analysis,$k_{A}$ is the Archard wear coefficients.

#### Energy model

The energy model considers that the wear volume is linearly related to the accumulated dissipated energy which is partially consumed to overcome the adhesive force of adhesive wear and the shearing force of abrasive wear, and the residual energy is dissipated in the form of heat or sound energy converted from frictional work, which is an empirical equation based on the experimental finding ^31^. In this model, the relationship between wear volume and the cumulated dissipated energy is defined as

4 $$ V = k_{e} E_{d} $$

where $E_{d}$ is the cumulated dissipated energy in J, $k_{e}$ is the energy wear coefficients, represented the wear volume generated by unit dissipated energy. According to Coulomb's friction law^31^, the cumulated dissipated energy can be expressed as

5 $$ E_{d} = fFL $$

which $f$ is the friction coefficient. Equation (4) can be re-written as Eq. (6).

6 $$ V = k_{e} fFL $$

Equation (6) can be divided by a contact area,

7 $$ \frac{dV}{{dLdA}} = k_{e} f\frac{dF}{{dA}} $$

Equation (7) can be re-written as Eq. (8) to implement in 3D FEA models.

8 $$ dh = k_{e} fp(x)dL $$

#### Wear coefficient

The Archard wear coefficients $k_{A}$ can be calculated by Eq. (9).

9 $$ k_{A} = \frac{V}{FL} $$

The energy wear coefficients $k_{e}$ can be calculated by Eq. (4), and the cumulated dissipated energy $E_{d}$ can be calculated by numerical integration of plots according to Fig. 2^32,33^. The Archard and energy wear coefficients for the reciprocating pin-on-plate tribological experiment and other related calculation parameters were shown in Table 2.

**Table 2:** Wear coefficients and other related calculation parameters.

Parameters	Magnitude		
The normal load *F* (N)	60		
The total accumulated slip distance *L* (mm)	360,000		
Disc surface roughness Ra(μm)	0.03	0.75	2.04
Wear volume(mm^3^)	0.32609	0.65217	1.521744
The Archard wear coefficients $k_{A}$(MPa^-1^)	1.50967 E−8	3.01931 E−8	7.03616 E−8
The energy wear coefficients $k_{e}$(MPa-1)	1.877E−7	3.753 E−7	8.758 E−7

## Finite element model

### Basic Assumptions

1. The viscoelasticity of the UHMWPE is ignored in finite element mode^19^;
2. The effect of temperature on UHMWPE properties is neglected in finite element model^16,17^;
3. The geometric model does not consider surface roughness and plastic deformation of the surface due to wear and third body between in the interface^11^;
4. Assuming that the wear coefficient for a friction pair under a condition is constant^8^.

### Geometry and configuration

**Figure 6:**
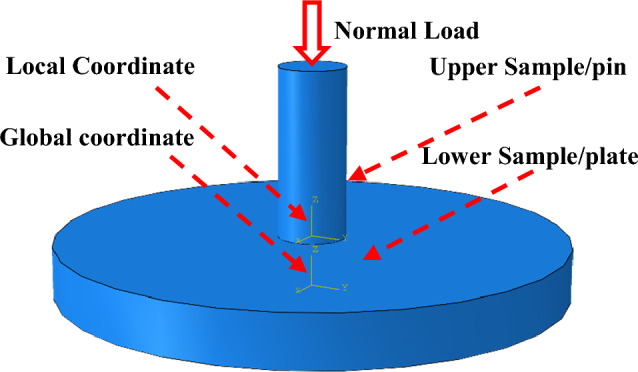
Wear finite element model.

According to the principle of a standard pin-on-plate tribometer, the wear finite element model of UHMWPE is established, which is primarily composed of upper and lower samples. The size of lower samples is the same as that of the plate in Table 1, and the size of the upper sample (pin) is with a 4-mm diameter and 10-mm length ignoring the length of the clamping part, as shown in Fig. 6. There are two coordinate systems which are parallel to each other in Fig. 6, one is the global coordinate system and the other is the local coordinate system of the pin. Since the hardness of the plate is notably greater than that of the pin (UHMWPE), considering that the material properties of the lower sample have little influence on the finite element model analysis results, it is assumed that the lower sample is a discrete rigid body. The constitutive model for UHMWPE is the elastic–plastic constitutive model, of which elastic behavior is linear elastic model (Hooke's law) and plastic behavior use Mises yield surface with associated plastic flow and isotropic hardening. Based on the tensile test results, the model parameters are shown in Table 3.

**Table 3:** The elastic–plastic constitutive model parameters.

Elastic modulus/MPa	280										
Poisson's ratio	0.4										
True stress/MPa	14.32	16.20	17.58	18.64	19.45	20.09	20.60	21.03	21.39	21.71	22.00
Plastic strain	0	0.015	0.029	0.040	0.052	0.064	0.076	0.088	0.10	0.11	0.12

### Boundary conditions

The contact relationship is established between upper and lower samples. The contact discretization is surface-to-surface. The finite-sliding formulation is used for the contact tracking approach. The bottom surface of the pin is defined as the slave surface, and the top surface of plates is defined as the master surface. The tangential behavior is defined as a penalty. The normal behavior is defined as a hard contact.

The analysis step settings are as follows: a normal load is applied to the upper end of the upper sample in the negative direction of the Z-axis in the first analysis step; next, the lower sample is reciprocated along the X-axis with a displacement of 5 mm following each step using the Python (V2.7.3) programming language, which refers to the global coordinate system. Each step time was 0.05 s, the total cumulative sliding distance was 360,000 mm, and the total step number was 72,000. The element type of the upper sample is C3D8.

### UMESHMOTION user-defined subroutine

UMESHMOTION is a user-defined subroutine for the commercial software ABAQUS that can define the movement of nodes in an adaptive mesh area. UMESHMOTION enables contact surface nodes to move in the local normal direction by defining contact surface nodes in the adaptive mesh constraint node-sets. The movement distance of the contact surface node in the local normal direction is the corresponding local wear depth, and the local normal direction free from outside interference. However, when the contact surface node is at the edge or corner, the movement direction of the node should be perpendicular to the contact surface, rather than along the local normal direction of the node. Therefore, it is necessary to calculate the wear direction in the UMESHMOTION subroutine. The specific method refers to the wear simulation of Sects. 3.1.8 of tire wear in reference^34^.

### Wear simulation procedure

**Figure 7:**
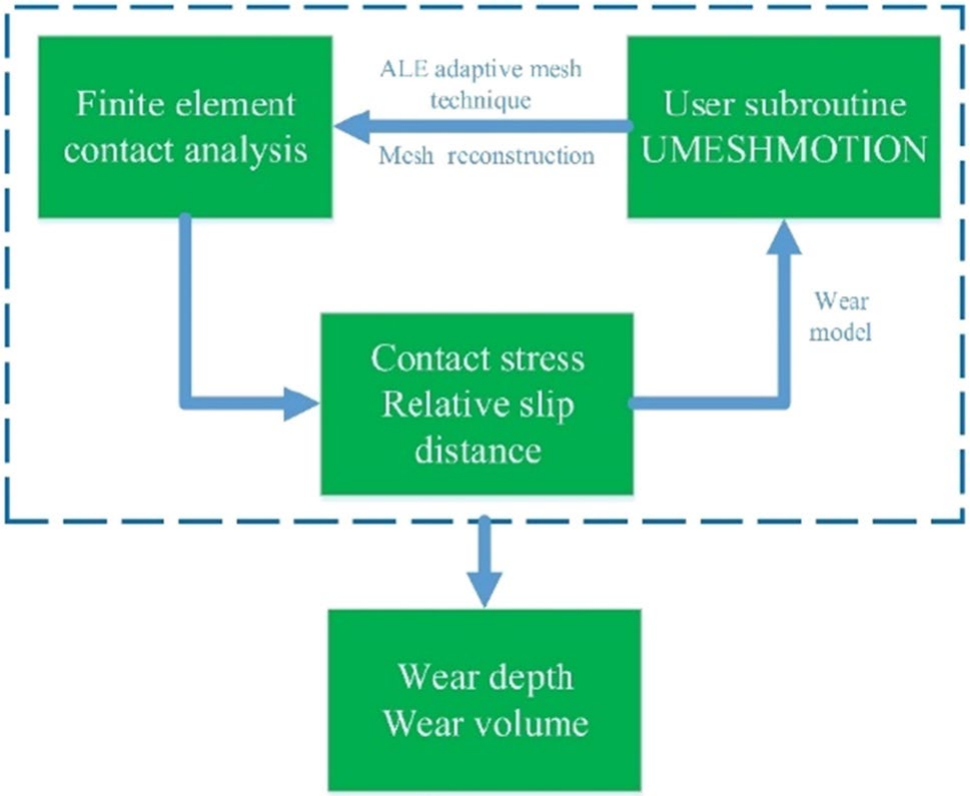
Flow chart of the wear simulation model.

The workflow chart of the wear simulation using UMESHMOTION is shown in Fig. 7. First, a 3D contact simulation model was established using ABAQUS to calculate the contact stress and cumulative sliding distance of contact surface. After the contact calculation convergence, each node on the contact surface calls the UMESHMOTION subroutine, which primarily feeds back the local wear increments for each node of the ABAQUS contact surface for a given time increment. ABAQUS' ALE adaptive mesh technology will perform mesh reconstruction in three steps based on the local wear increment of all surface nodes. In the first step, the surface nodes scan along the local normal direction to redefine the node position, and the scanning displacement along the local normal direction is equal to the corresponding local wear increment. The scanning of the nodes is performed as a Euler analysis, updating only the geometry. In the second step, the second-order numerical method (Lax-Wendroff method) is used to solve the mesh smoothing equation and move the material from the old position to the new position. Finally, ABAQUS iteratively solves the contact problem again, correcting the balance loss caused by the scanning of the mesh and the movement of the material, and updating the contact stress. The above process is repeated until the analysis time is reached^34^.

### Model convergence analysis

**Figure 8:**
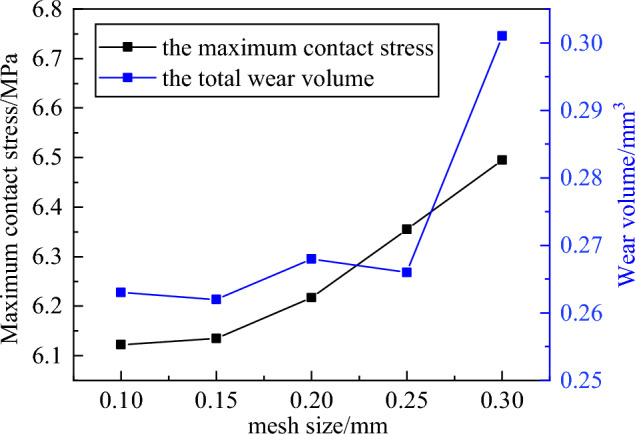
Model convergence analysis.

**Figure 9:**
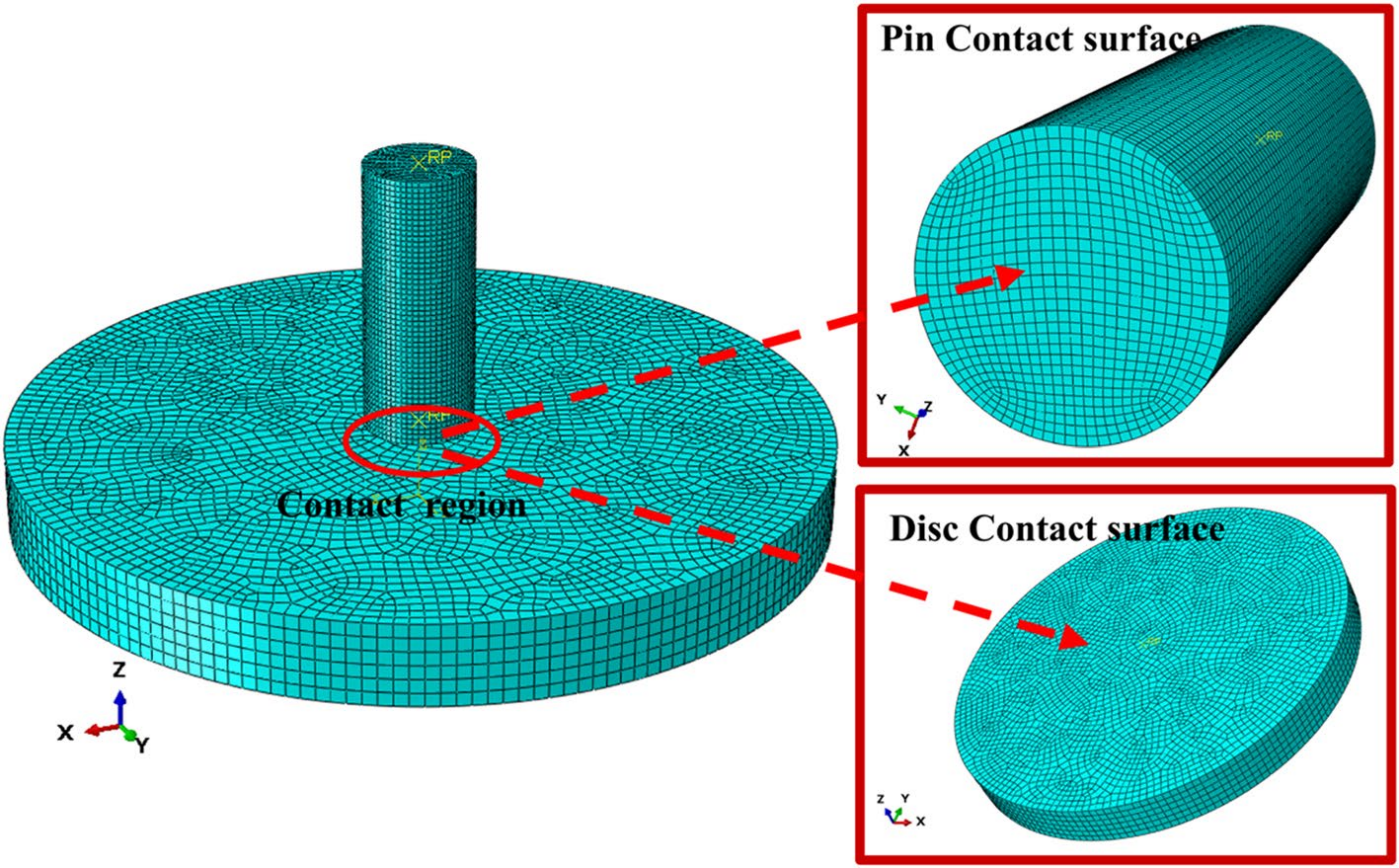
Mesh nodes distribution of the finite element model and contact region.

As is well-known, the appropriate mesh density is very important for finite element analysis, which can reduce the influence of the boundary effect and directly determine the accuracy of finite element analysis results^35^. When finite element model is used to simulate the wear, the sensitivity analysis of the unworn model and the worn model should be carried out^36^. In this paper, the maximum contact stress on the contact surface in pin after first analysis step is the sensitivity index of unworn model. The wear volume of the pin against plate with surface roughness 0.03 μm is sensitivity index of worn model, of which wear simulation finite element model is based on Archard model. Mesh size is changed with a certain rule until the maximum contact stress value of the unworn model and the total wear volume of the worn model remain largely unchanged. Finally, the mesh size is determined with less computation time and cost as the criterion. The mesh refinement schemes and results used for two mesh sensitivity analyses are displayed in Fig. 8. After model convergence analysis, the mesh size is determined to be 0.15 mm with total mesh numbers 46096, and mesh nodes distribution of the finite element model and contact region is shown in Fig. 9. The overall calculation time is approximately 56 h.

## Results and discussion

### Contact stress distribution

**Figure 10:**
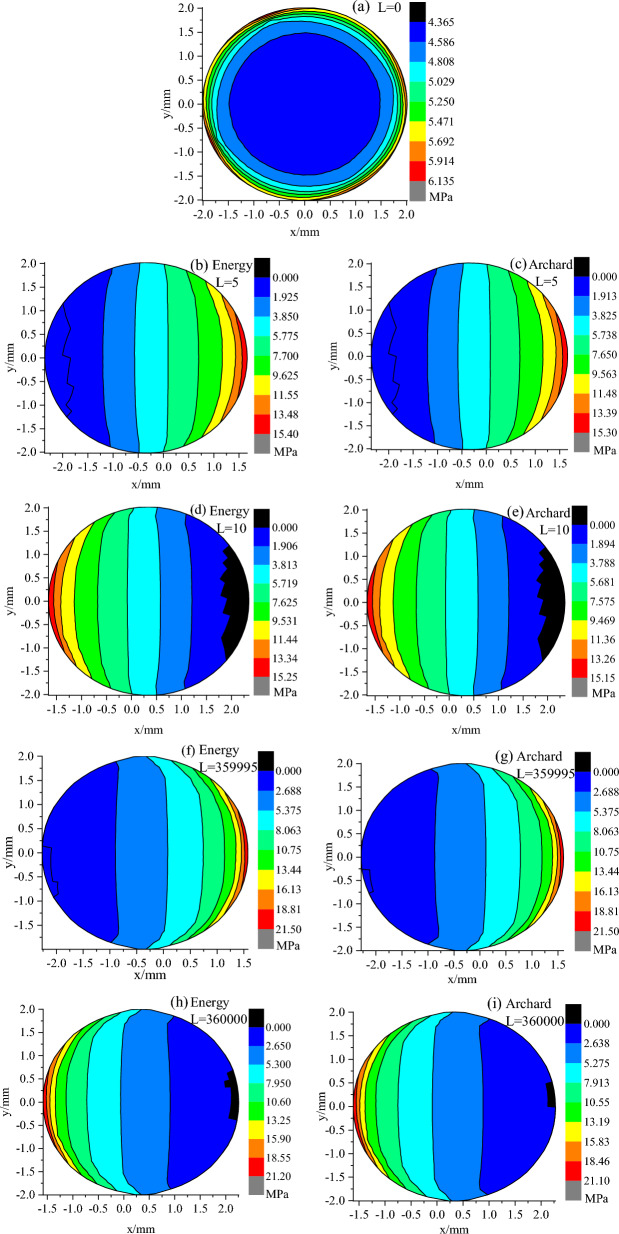
Distributions cloud of contact stress (**a**–**i**) in the worn surface of pin against plate with surface roughness Ra 0.03 μm.

Figure 10 shows the distributions of contact stress in worn surface of the pin against the plate with Ra 0.03 μm based on the Archard model and the Energy model, respectively, where *L* represents the cumulative sliding distance. During reciprocating motion, it could be observed that the region with contact stress is zero and the region with large contact stress appeared alternately along the direction of reciprocating motion (X-axis).

**Figure 11:**
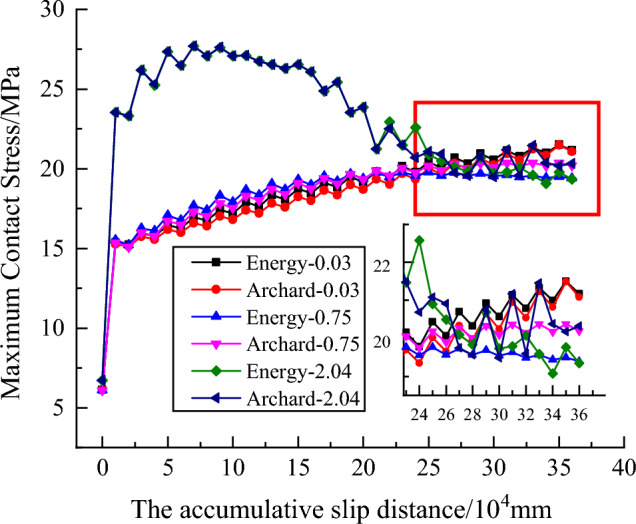
Maximum contact stress change curve.

Figure 11 reveals the maximum contact stress change rule in the worn surface of the pin against the plate with various roughness values based on the Archard model and energy model, respectively, during reciprocating motion. As the cumulative sliding distance increased, the maximum contact stress first increased and then stabilized. The maximum contact stress rapidly rises for all six cases at the beginning. While for the plate with a roughness of 2.04 μm it increased to the peak first and then decreased over the first 250,000 mm cumulative sliding distance at a faster rate, followed by a gradual decrease but at a slower rate; for the plate with a roughness of 0.75 μm it increased to the peak first and then decreased at a slower rate with energy model, but for the Archard model it seems to increase during the cumulative sliding distance of 360,000 mm, while the difference is little; for plate with a roughness of 0.03 μm it increases the entire time at a slower rate.

Compared with others, fluctuations of the maximum contact stress in the worn surface of the pin against the plate with a roughness of 2.04 μm were obvious during the 250,000 mm cumulative sliding distance. These results suggest that the plate surface roughness had a significant influence on the maximum contact stress and the wear model had minimal influence on the maximum contact stress.

### Wear depth distribution

**Figure 12:**
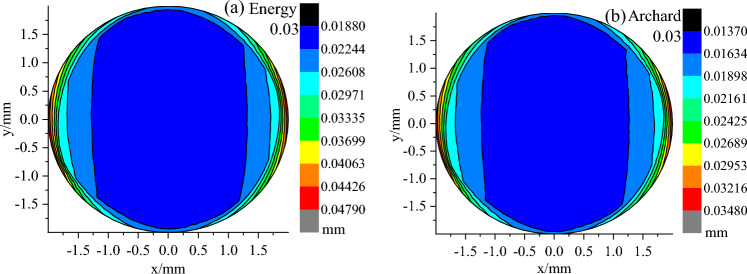
Distribution of wear depth with 0.03 μm roughness calculated by the energy model and Archard model.

**Figure 13:**
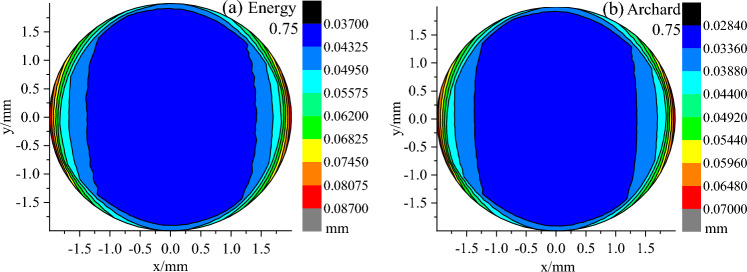
Distribution of wear depth with 0.75 μm roughness calculated by the energy model and Archard model.

**Figure 14:**
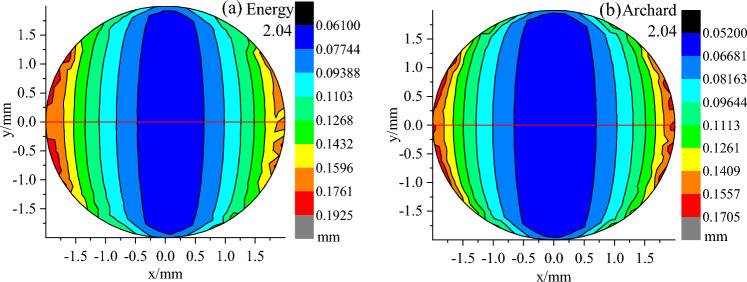
Distribution of wear depth with 2.04 μm roughness calculated by the energy model and Archard model.

Figures 12, 13 and 14 show the wear depth distribution clouds on the worn surface of the pin against it with 0.03, 0.75 and 2.04 μm roughness using the Archard model and the energy model to calculate them, respectively. The wear depth is a scalar. Along the direction of reciprocating motion, the wear depth was larger on both sides of the pin and was smaller in the middle. This is attributed to the distribution of contact stress during reciprocating sliding.

**Figure 15:**
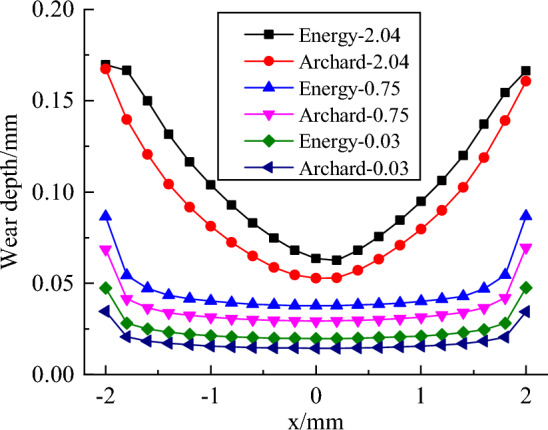
Wear depth distributions on the cross section of y = 0 with various surface roughness calculated by the energy model and Archard model.

In addition, the maximum and minimum wear depth increased with the increase in surface roughness, and the maximum and minimum wear depth calculated by the Energy model was larger than that of the Archard model under the same roughness. To further determine the influence of the two models on the wear depth distribution, the depth values on the y = 0 section (the red line in Fig. 15) along the X-axis were extracted from the wear depth distribution cloud of each surface roughness calculated by the two models, as shown in Fig. 15. With the increase of surface roughness, the wear depth increased, and at the same roughness, the wear depth calculated by the energy model is larger than Archard's along the X-axis. In contrast to the plate with a surface roughness of 2.04 μm, the wear depth distributions with a surface roughness of 0.75 and 0.03 μm were similar, except for different depth values.

### Wear profiles

**Figure 16:**
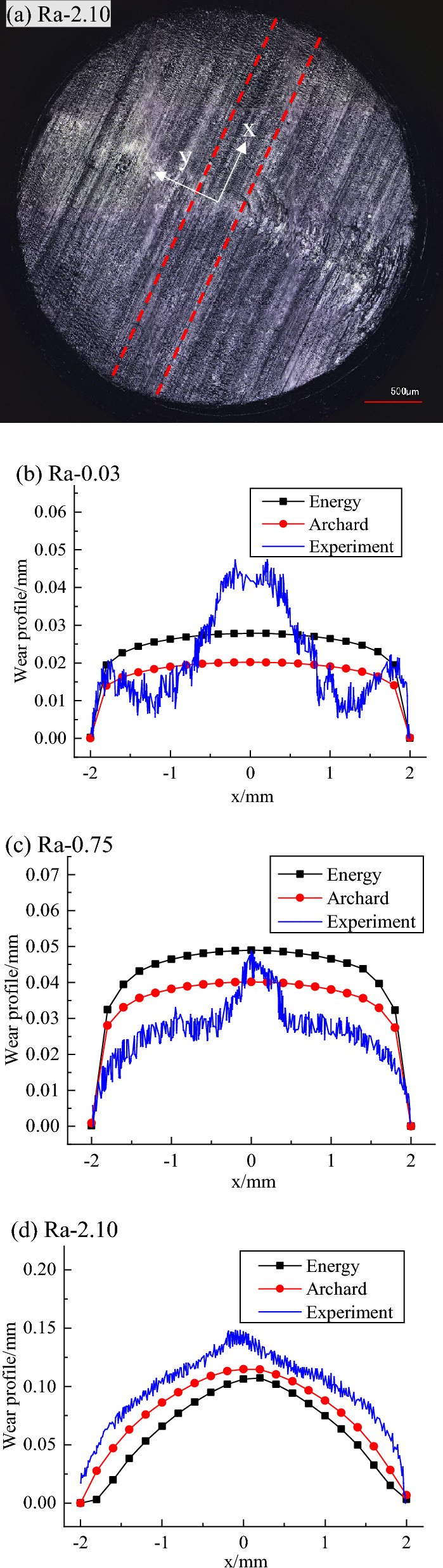
Optical micrographs of the overall worn surface of pin (**a**) against plates with 2.04 μm surface roughness and comparison of finite element simulation and experimental results for worn profiles of the of pins against plates with 0.03-μm (**b**), 0.75-μm (**c**) and 2.04-μm (**d**) surface roughness.

To compare the experimental results with the finite element simulation results, the 3D profilometric scans were used to scan the worn contour of pins. The wear profiles in the middle of the pins were obtained by extracting 5 groups of wear profile data along the reciprocating sliding direction and then calculating the average value, which is shown in Fig. 16. It refers to the coordinate system of which the XY plane is the same as the local coordinate system (Fig. 6), the Z axis direction is the opposite of the Z axis of the local coordinate system and the origin is located at the center of the XY plane which has the lowest point of the worn profile. Figure 16a shows the overall surface morphology of the pin against the plate with a surface roughness of 2.04 μm after a 60 min wear test, where the red line represents the token track of the worn contour along the direction of reciprocating motion. The worn surface profiles of pins against plates with various surface roughness values, calculated by the energy model and Archard model have been compared with experimental results for all three cases and are presented in Fig. 16b–d. It is clear that except for a surface roughness of 0.03 µm, the trend of the wear profile distribution obtained by simulation was similar to the case of the experimental results.

The reasons for the simulation results in the presence of the plate with a surface roughness 0.03 μm being different from the wear profile distribution of the experimental results can be explained by the fact that the wear volume is small, and then the influence of the initial surface roughness observed markedly in Fig. 4b, in which the peak height or valley depth may be larger than local wear depth at some position, is significant, and it is not considered in the current finite element models.

### Wear volume

**Figure 17:**
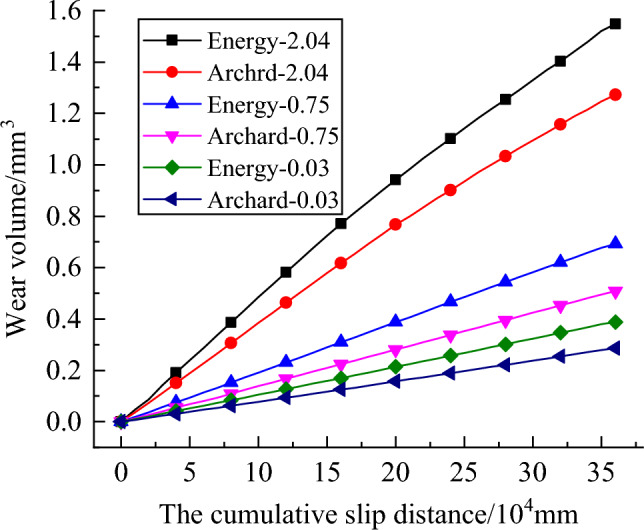
Evolution of wear volume using the Archard model and energy model under various counterface roughness.

In Fig. 17, it is found that as the sliding distance increases, the wear volume of the pin increases, the volume predicted by the energy model is larger than the volume predicted by the Archard model, and the difference becomes larger and larger, especially in the case of plate surface roughness of 0.75 μm, where the difference is the greatest.

**Figure 18:**
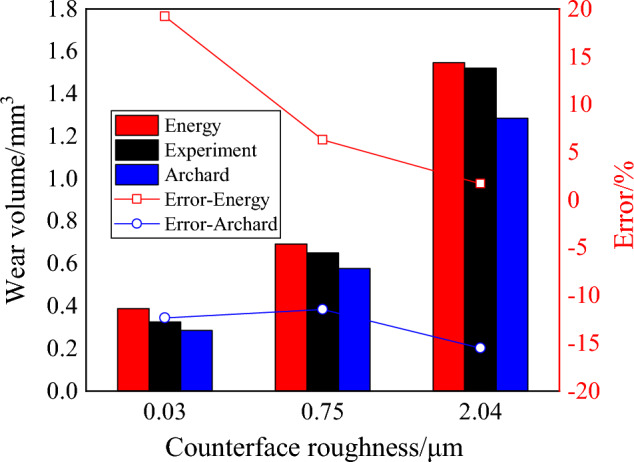
Wear volume and its error between the simulation and experimental results.

In addition, there are significant differences between the results obtained by the two wear models and the experimental result, but the relative error of wear volume between the finite element simulation of two models and experiment is within 20%, which was presented in Fig. 18. Compared with experiment results, the wear volume estimated by the Energy model is overestimated, and the relative error value decreases with the increase in surface roughness of plate. With respect to the wear volume estimated by the Archard model, it is underestimated. When the surface roughness of the plate is 0.03 μm, the relative error value of the Energy model is larger than that of the Archard model. When the surface roughness of the plate is greater than 0.75 μm, the relative error value of the Energy model is less than that of the Archard model. Particularly in the condition that surface roughness equals 2.04 μm, the relative error value of the Archard model is up to 16%, and the relative error value of the Energy model is as low as 1.7%. According to the above analysis, the wear volume obtained by the Energy model and experiment results were found to best fit, when the surface roughness of the plate is greater than 0.75 μm.

The reasons for these differences between the results of the two models may be attributed to the wear model itself. With the increase in the surface roughness of the plate, the more complex the friction interaction between the UHMWPE pin and plate is in the wear process, which includes the material structure transformation, chemical and physical processes, and debris behavior found in section "Tribological tests". However, the Archard model is mainly used to describe adhesive wear or cutting without considering the complex friction interaction; therefore, it may cause error when the wear mechanism is complex (Supplementary Information).

## Conclusions

This study focused on two points: (1) the possibility of the 3D finite element model using the UMESHMOTION subroutine developed by ABAQUS software to simulate the wear of UHMWPE; (2) the adaptability of energy model and Archard model to simulate the wear of UHMWPE against the different counterface roughness of which the study range is 0.03 ~ 2 μm in reciprocating unidirectional sliding motion in FE method. The FE wear models of tribological tests were respectively established based on the Archard model and energy model to calculate the wear of UHMWPE against the different counterface roughness. The adaptability of two models were investigated by analyzing the microscopic morphology and the error of wear profiles and wear volume between the experiment and simulation. The current study revealed the following conclusions:

1. The distribution rules of the worn profiles estimated by the finite element wear models were similar to the experiment results, and the error of wear volume between the experiment and simulation is within 20%, which largely verifies the possibility of 3D finite element model using the UMESHMOTION subroutine developed by the ABAQUS software to simulate the wear of UHMWPE in reciprocating unidirectional sliding motion.
2. With the increase in the counterface roughness, the wear mechanism of UHMWPE becomes more complex and the wear volume and worn profiles predicted by the energy model is more accuracy, which may prove that the energy model can provide more physical explanations for the wear of UHMWPE in reciprocating unidirectional sliding motion.
3. In this paper, viscoelasticity of the UHMWPE and effect of temperature on UHMWPE properties is neglected in finite element model, which will affect the accuracy of the calculation results. In addition, this paper only discusses the wear of UHMWPE in reciprocating unidirectional sliding motion, and the conclusion has some limitations. We will continue to be improved in the next research.

### Supplementary Information

Supplementary Information.

## Data Availability

The experiment data generated during this study are included in this published article and its supplementary information files, and the others datasets used and/or analysed during the current study available from the corresponding author on reasonable request.
